# A Biocompatible Ultrananocrystalline Diamond (UNCD) Coating for a New Generation of Dental Implants

**DOI:** 10.3390/nano12050782

**Published:** 2022-02-25

**Authors:** Orlando Auciello, Sandra Renou, Karam Kang, Deborah Tasat, Daniel Olmedo

**Affiliations:** 1 Department of Materials Science and Engineering and Bioengineering, Original Biomedical Implants (OBI-USA), University of Texas at Dallas, Dallas, TX 75080, USA; oha120030@utdallas.edu; 2 Original Biomedical Implants-México (OBI-México), Hermosillo 83210, Mexico; 3 Cátedra de Anatomía Patológica, Facultad de Odontología, Universidad de Buenos Aires, Buenos Aires C1122AAH, Argentina; sandra.renou@odontologia.uba.ar; 4 Hoefer Welker, Dallas, TX 75201, USA; karam0225@gmail.com; 5 Cátedra de Histología y Embriología, Facultad de Odontología, Universidad de Buenos Aires, Buenos Aires C1122AAH, Argentina; dtasat@gmail.com; 6 Escuela de Ciencia y Tecnología, Universidad Nacional de General San Martín, San Martín 1650, Argentina; 7 Instituto de Tecnologías Emergentes y Ciencias Aplicadas (ITECA), CONICET—Universidad Nacional de General San Martín, San Martín 1650, Argentina; 8 Consejo Nacional de Investigaciones Científicas y Técnicas (CONICET), Buenos Aires C1425FQB, Argentina

**Keywords:** ultrananocrystalline diamond (UNCD) coating, surface treatment, biotribocorrosion, biocompatibility, titanium micro-implants, osseointegration

## Abstract

Implant therapy using osseointegratable titanium (Ti) dental implants has revolutionized clinical dental practice and has shown a high rate of success. However, because a metallic implant is in contact with body tissues and fluids in vivo, ions/particles can be released into the biological milieu as a result of corrosion or biotribocorrosion. Ultrananocrystalline diamond (UNCD) coatings possess a synergistic combination of mechanical, tribological, and chemical properties, which makes UNCD highly biocompatible. In addition, because the UNCD coating is made of carbon (C), a component of human DNA, cells, and molecules, it is potentially a highly biocompatible coating for medical implant devices. The aim of the present research was to evaluate tissue response to UNCD-coated titanium micro-implants using a murine model designed to evaluate biocompatibility. Non-coated (*n* = 10) and UNCD-coated (*n* = 10) orthodontic Ti micro-implants were placed in the hematopoietic bone marrow of the tibia of male Wistar rats. The animals were euthanized 30 days post implantation. The tibiae were resected, and ground histologic sections were obtained and stained with toluidine blue. Histologically, both groups showed lamellar bone tissue in contact with the implants (osseointegration). No inflammatory or multinucleated giant cells were observed. Histomorphometric evaluation showed no statistically significant differences in the percentage of BIC between groups (C: 53.40 ± 13% vs. UNCD: 58.82 ± 9%, *p* > 0.05). UNCD showed good biocompatibility properties. Although the percentage of BIC (osseointegration) was similar in UNCD-coated and control Ti micro-implants, the documented tribological properties of UNCD make it a superior implant coating material. Given the current surge in the use of nano-coatings, nanofilms, and nanostructured surfaces to enhance the biocompatibility of biomedical implants, the results of the present study contribute valuable data for the manufacture of UNCD coatings as a new generation of superior dental implants.

## 1. Introduction

Pure titanium (Ti) and its alloys are widely used to manufacture dental and orthopedic implants, among other medical applications, given their appropriate mechanical properties and biocompatibility [1,2]. Osseointegratable Ti dental implants have revolutionized clinical dental practice and have shown a high rate of success [3,4]. However, some technical and/or biological complications associated with Ti dental implants can occur [5,6].

No metal or metal alloy is completely inert in vivo [7]. One of the possible causes of failure of a Ti implant after initial success is biotribocorrosion, which is the combined effect of mechanical, biochemical, and electrochemical factors in a biological environment [8,9,10]. Biotribocorrosion causes the release of ions/metallic particles from the Ti surface into the surrounding tissue, thus allowing for their possible systemic dissemination and deposition in distant organs [11].

Several studies have reported the presence of Ti particles in human peri-implant tissues [6,9,12]. In line with studies reported in the literature, our research group histologically demonstrated the presence of Ti particles in peri-implant tissue surrounding failed human dental implants [13], in oral mucosa in contact with the implant cover screw [14], in cells exfoliated from the oral mucosa in contact with a titanium dental implant [15], and in lesions found close to a Ti dental implant [16,17].

In situ degradation of a metallic implant alters its structural integrity, and the released ions/particles can have different biological effects [7,18,19,20,21,22]. Aside from creating new metal-free implant materials that can be used for oral rehabilitation purposes, it is paramount to design enhanced implant surface treatments to minimize the risk of implant biotribocorrosion and the health problems associated with these processes. A number of coatings and micro- and nanostructured surfaces have been developed in an attempt to improve implant biocompatibility and osseointegration and prevent the release of ions/particles from the implant surface into the biological milieu. The properties of these coatings are evaluated using different biological parameters [23,24].

An ideal surface treatment would render the coating highly resistant to chemical attack and would allow achieving micro- and nano-roughness, which has been shown to enhance osseointegration [25,26]. Nanoscale-tailored surfaces can have a more profound and significant impact on the fates of cells compared with microscaled surfaces [27,28,29]. A number of studies have engineered nanotopological features including nanogrooves, nanofibers, nanodots, nanotubes, and complex shaped patterns [30] and have investigated the significant effects of nanostructured implant surfaces on osteogenic differentiation and the immune system [31].

Carbon-based materials [32,33] have emerged as promising candidates for implant coatings in view of their good tissue compatibility, resistance to chemical attack by body fluids, radiation resistance (which makes them suitable for sterilization processes), hemocompatibility, optimal mechanical and tribological properties (ultrahigh hardness, low friction coefficient, negligible wear), and good adhesion to Ti [34].

The ultrananocrystalline diamond (UNCD) coating described in this article is one of the carbon coatings available today and was developed [35,36,37,38,39,40] and patented [38,41] by Auciello et al. This novel UNCD coating exhibits a unique synergistic combination of outstanding mechanical (highest hardness compared to all other carbon-based coatings [37]) and tribological (lowest coefficient of friction compared to all other carbon-based coatings [36,37]) properties, unique resistance to chemical corrosion by any fluid [37] (including body fluids [40]), high electrical conductivity when inserting N atoms in the grain boundaries providing electrons for conduction or B atoms replacing C atoms in the diamond grain lattice and providing electrons to the electric conduction band [37,42], and exceptional biocompatibility properties [23,37,40,43]. More specifically, its hardness (98 GPa) [37] is similar to that of a diamond gem (100 GPa) and thus greater than that of any other material in thin film form. The UNCD coating exhibits an extremely high fracture resistance/coefficient and practically no wear and has one of the lowest coefficients of friction (~0.02–0.04) compared to coatings available today for industrial products (mechanical pump seals and bearings [37]) and implantable prostheses (hips, knees, and so forth) for clinical use. UNCD is extremely resistant to chemical corrosion by any fluid, including strong acids like HF and body fluids.

As compared to other types of diamond-based coatings, the surface structure of UNCD films facilitates adhesion, proliferation, and metabolism of different cell types, and thus resembles the nanoscale extracellular matrix of tissue [44]. As shown by studies evaluating the interaction between UNCD films and osteoblasts, fibroblasts, cortical neurons, and cortical stem cells, UNCD films are a suitable, non-toxic support surface for cell growth and proliferation [45,46,47,48,49].

Nanocrystalline diamond (NCD) and UNCD coatings have also been shown to be useful for biosensing post-surface functionalization [50,51,52]. Auciello et al. showed the UNCD coating to be an extremely biocompatible/eye-fluid-corrosion-resistant coating to encapsulate a Si microchip (artificial retina), implantable inside the human eye to restore partial vision to people blinded by retinitis pigmentosa (genetically induced degeneration of retina photoreceptors) [23]. Other researchers have evaluated the use of other diamond structures [53,54] and UNCD coatings [55] for ophthalmological devices, focusing on restoring sight to patients with retinitis pigmentosa. UNCD has been proposed as a coating for biomedical devices, such as coxofemoral prostheses, dental implants, cardiac valves, and ocular devices [23,36].

UNCD can be micromachined to produce tailored micro-nano-electro-mechanical systems (MEMS/NEMS) [35], such as biosensors and drug delivery devices; all these systems are based on UNCD films [37]. UNCD films are grown using Microwave Plasma Chemical Vapor Deposition (MPCVD) or Hot Filament Chemical Vapor Deposition (HFCVD). The HFCVD method currently yields more homogenous films on large areas (up to 300 mm in diameter) and is being used for manufacturing commercial industrial products such as UNCD-coated mechanical pump seals and bearings developed by Advanced Diamond Technology, a company founded by Auciello and colleagues in 2003 (www.thindiamond.com, accessed on 31 December 2021). UNCD thin films grown on metallic, semiconductor, and insulating surfaces exhibit an inherently smooth topography (~7–10 nm rms roughness). Auciellos’ group demonstrated that a nanometer-scale-thick tungsten (W) layer (~50–100 nm) grown on the surface of any substrate used to grow UNCD films induces much denser and smoother (~3–5 nm rms surface roughness) UNCD films than films grown without the W layer [39].

In view of the mechanical and chemical properties of UNCD coatings, the aim of this study was to evaluate tissue response to UNCD-coated Ti micro-implants using a murine model to assess the biocompatibility of the UNCD coating.

## 2. Materials and Methods

### 2.1. Implants

**Figure 1:**
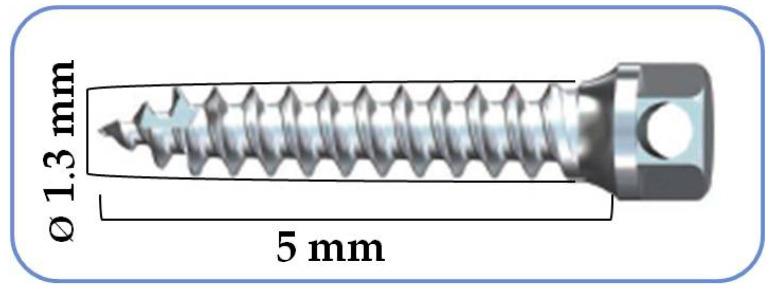
Schematic drawing of a micro-implant (AbsoAnchor^®^ NH 1312-05, Dentos Inc., Daegu, Korea) showing implant size.

Orthodontic Ti micro-implants 5 mm in length and 1.3 mm in width were used (Ti-6Al-4V, AbsoAnchor^®^ NH 1312-05, Dentos Inc, Daegu, Korea) (Figure 1). The micro-implants were assigned to one of two groups: a Control group (C), which included non-coated micro-implants (*n* = 10), and an Experimental group (E) consisting of UNCD-coated micro-implants (*n* = 10).

### 2.2. Method to Grow a Tungsten (W) Interface Layer on Ti Micro-Implants

A tungsten (W) interface layer was grown on the Ti micro-implant surface using conventional RF magnetron sputtering. Magnetron sputter-deposition was carried out using an input RF power of 150 W and an Ar gas flow of 30 sccm (mTorr-range pressure) at room temperature to strike a plasma discharge on the surface of a solid W target in the magnetron system. The Ar ions impacting on the surface of the W target induce ejection of W atoms that travel across the space between the target and the Ti substrate to land on the Ti surface and induce the growth of the W layer.

### 2.3. Method to Grow a UNCD Coating on W-Coated Ti Micro-Implants

The UNCD film was grown on the surface of W-coated Ti micro-implants employing the Microwave Plasma Chemical Vapor Deposition (MPCVD) technique using a commercial IPLAS (Innovative Plasma Systems GmbH, Troisdorf, Germany) MPCVD system. The novel patented UNCD growth process involves introducing an Ar-rich (99%)/CH4 (1%) gas mixture into the MPCVD chamber, evacuated of air to produce a base pressure of ~5 × 10^−7^ Torr. A mixture of Ar (49.2 sccm)/CH4 (0.8 sccm) gas was flown into the evacuated chamber producing a pressure of 90 mbar. Microwave power (1200 watts) was coupled onto the gas, producing a plasma that generates C2-dimers (main UNCD nucleation species), CH_3_, CH_2_, and CH radicals, which induce the growth of the UNCD films upon impacting on the substrate surface [37]. UNCD films were grown on heated (800 °C) Ti implant samples coated with a ~100 nm W layer with the surface seeded with nanocrystalline diamond particles embedded on the W surface by ultrasound waves in an ultra-sonicator with a solution of nano-diamond particles in methanol. After the initial seeding, extensive surface cleaning involving sequential exposure of the surface to solvents was performed to remove all large particulates from the substrate surface prior to film growth. UNCD films were subsequently grown for different periods of time to achieve the desired film thickness (~0.5–1 µm).

### 2.4. Physical-Chemical Characterization of the Implant Surface

Samples of micro-implants from both groups were examined using scanning electron microscopy (SEM Zeiss Supra model 40, Oberkochen, Germany). For SEM imaging, the samples were coated with a thin (20 nm) layer of silver in a vacuum evaporator to make them electrically conductive and enable optimum SEM imaging. In addition, the chemical composition of the samples was determined using Energy-dispersive X-ray Spectroscopy (EDS, INCAx-sight model, Oxford Instruments, High Wycombe, UK), with an analytical least detectable dose of 0.5%.

### 2.5. Experimental Animals

Male Wistar rats, 120 g body weight, were used. The animals were housed under standard conditions, receiving water and food ad libitum and under 12:12 light–dark cycles and controlled temperature (22–24 °C) conditions. The protocol was approved by the Institutional Experimentation Committee (School of Dentistry of the University of Buenos Aires, Resolution Number 006/2015). Adequate measures were taken to minimize animal pain and discomfort. All procedures were performed in compliance with the National Institutes of Health (NIH) guidelines for the care and use of laboratory animals (NIH Publication—Guide for the Care and Use of Laboratory Animals: Eighth Edition, 2011) and the guidelines of the School of Dentistry of the University of Buenos Aires (Res. (CD) 352/02 and Res. (CD) 694/02).

### 2.6. Surgical Procedure

The animals were anesthetized by intraperitoneal injection of a solution of 8 mg of ketamine chlorhydrate (Fort Dodge^®^, La Plata, Provincia de Buenos Aires, Argentine) and 1.28 mg of xylazine (Bayer, Leverkusen, Germany) per 100 mg of body weight. Implantation inside the medullary cavity of the tibiae was performed following the technique described by Cabrini et al. [56]. A non-coated micro-implant was placed inside the medullary compartment of the left tibia (C Group) and a UNCD-coated micro-implant was placed inside the medullary compartment of the right tibia (UNCD Group). Both lower limbs were shaved using an electric shaver (Philips^®^, Buenos Aires, Argentina), and a 1.5 cm incision was made along the tibial crest using a surgical blade (N° 11 Bard Parker^®^). The muscles and periosteum were dissected to expose the metaphyseal and diaphyseal region of the outer side of the tibia. A 1.5 mm hole was drilled through the bone by rotating a round burr (Dentsply Maillefer, Tulsa, OK, USA) manually to avoid heating and ensuing necrosis of the bone tissue. Each implant was placed inside the hematopoietic bone marrow, parallel to the longest axis of the tibia. The tissues were repositioned, and the skin was sutured with separate stitches (Vicryl^®^ N°3.0, Johnson & Johnson, New Brunswick, NJ, USA). No antibiotic therapy was administered. Thirty days post implantation, all the animals were euthanized by anesthetic overdose, and the tibiae were resected, fixed in 10% buffered formalin, and radiographed.

### 2.7. Histological Processing

The tibiae were processed and embedded in acrylic resin. Longitudinal histological sections were obtained using the micro-grinding system EXAKT 300 CP & 310 CP Precision Parallel Control (EXAKT, Hamburg, Germany) and stained with toluidine blue 1% for histological examination by light microscopy.

### 2.8. Histomorphometry

The histological sections were analyzed histomorphometrically using an optical photomicroscope (Leica, DM 2500, Wetzlar, Germany) with LAS EZ software (Leica Application Suite, Wetzlar, Germany) at a magnification of 400×. The area of peri-implant bone tissue and percentage of osseointegration (bone-implant contact: BIC) were determined.

### 2.9. Statistical Analysis

The data were statistically analyzed using Student’s *t*-test. Values are expressed as mean and SD; statistical significance was set at *p* < 0.05.

## 3. Results

### 3.1. Growth of W Interface Layer

The W layers grown by magnetron sputter-deposition were about 50 to 10 nm thick and had a surface roughness of about 6 nm rms, as measured using Atomic Force Microscopy (AFM). Scanning Electron Microscopy (SEM) and AFM studies showed that UNCD films grown on a substrate surface without the W layer exhibited a surface roughness of about 20 nm rms, while UNCD films grown on a 100 nm thick W layer showed a surface roughness of about 6.3 nm rms.

### 3.2. Growth of the UNCD Coating

**Figure 2:**
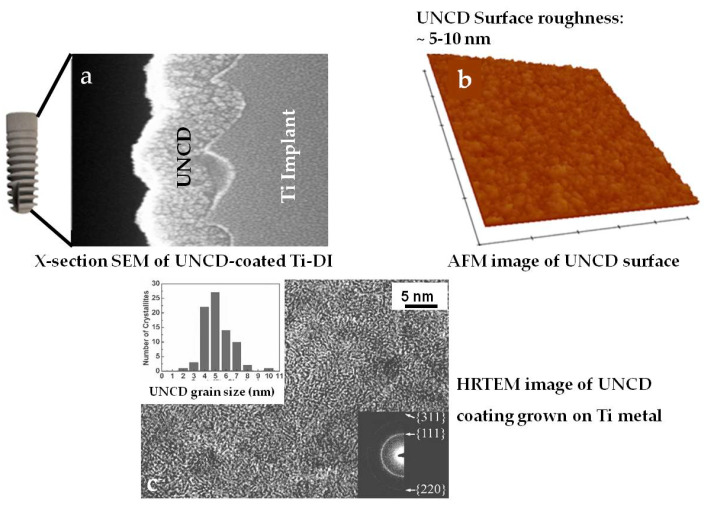
(**a**) Cross-section SEM image of a UNCD coating grown on a Ti micro-implant (note the extremely dense/conformal structure of the UNCD coating with a nanoscale surface roughness on the Ti implant); (**b**) surface roughness of the UNCD coating measured by Atomic Force Microscopy (AFM); (**c**) High-Resolution Transmission Electron Microscopy (HRTEM) image of the UNCD grown on a Ti micro-implant sample showing the typical UNCD grain size of 3–5 nm (the insert shows the electron diffraction pattern obtained in the HRTEM study, showing the (111), (311), and (220) rings characteristic of the diamond structure with nanograins).

UNCD coatings grown on the characteristic screw-type dental implants using the MPCVD process exhibited an extremely dense/pin-hole-free structure conformal to the screw-type implant, as shown by SEM (Figure 2a). Figure 2b shows the surface roughness of the UNCD coating as measured by the well-known Atomic Force Microscopy (AFM) technique. The AFM technique involves scanning a sharp metal tip, integrated in a cantilever, over the surface of the sample. A laser beam reflecting from the top surface of the cantilever is directed to a sensor, producing the image of a surface with atomic scale resolution. HRTEM studies showed that the UNCD films grown on Ti exhibited the typical grain size of 3–5 nm (Figure 2c).

### 3.3. SEM Imaging and EDS Analyses of Uncoated and UNCD-Coated Ti Micro-Implants

**Figure 3:**
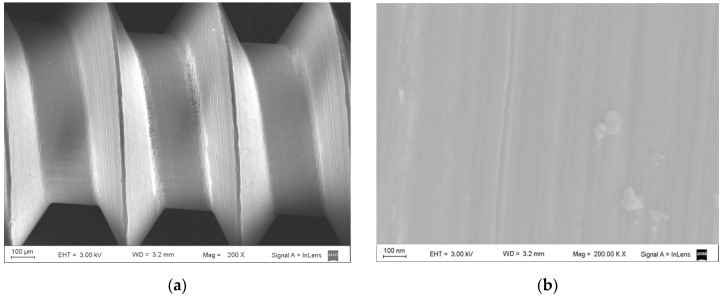
SEM images showing the surface features of a non-coated Ti micro-implant: (**a**) low-magnification (×200) SEM image showing the thread structure of the screw-type micro-implant; (**b**) high-magnification (×200,000) SEM image of the machined surface.

**Figure 4:**
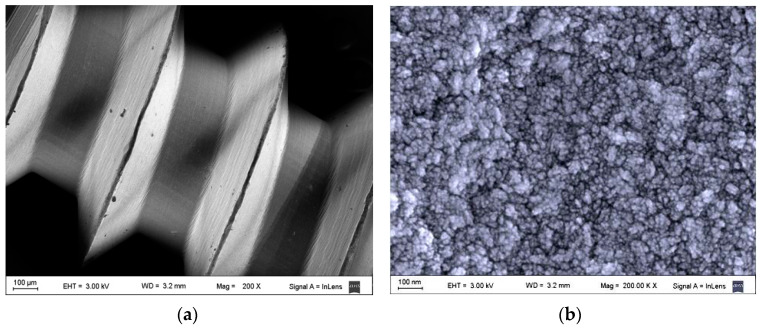
SEM images showing the surface features of a UNCD-coated Ti implant: (**a**) low-magnification (×200) SEM image showing the thread structure of the screw-type Ti micro-implant; (**b**) high-magnification (×200,000) SEM image of the UNCD-coated machine-finished Ti surface, showing the nanoscale grainy surface of the UNCD coating.

As shown by SEM, the surface of the uncoated Ti micro-implants in Group C showed all the typical features of a machine-finished surface (Figure 3a,b). The surface of the micro-implants in the UNCD group, however, was grainy, with grain sizes ranging from 3–5 nm (Figure 4a,b), and the coating fully covering the Ti surface of the implant.

**Figure 5:**
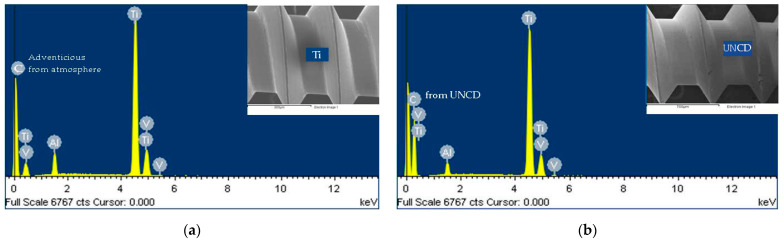
EDS analysis of micro-implants: (**a**) spectrum showing peaks corresponding to Al, Ti, and V in control micro-implants (the C peak arises from C contaminants adsorbed on the surface exposed to the atmospheric environment, as occurs with any material before insertion in the SEM vacuum system); (**b**) spectrum showing peaks corresponding to C, Al, Ti, and V in UNCD-coated implants.

The elemental composition of the micro-implants was qualitatively confirmed by EDS analysis. Composition of control micro-implants was 6.55% aluminum (Al), 90.43% titanium (Ti), and 3.03% vanadium (V), whereas UNCD-coated micro-implant composition was 42.77% carbon (C), 1.96% aluminum (Al), 54.60% titanium (Ti), and 0.68 vanadium (V) (Figure 5a,b).

### 3.4. Radiographic Study

**Figure 6:**
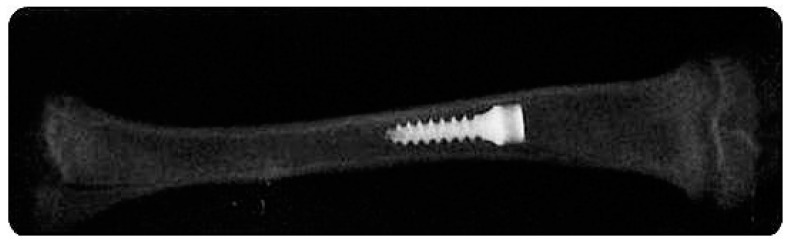
Radiograph showing a microimplant in place in the diaphysis of the rat tibial marrow cavity.

Radiographic evaluation revealed that all implants remained in the implantation site in the diaphyseal area (Figure 6).

### 3.5. Histologic Evaluation

**Figure 7:**
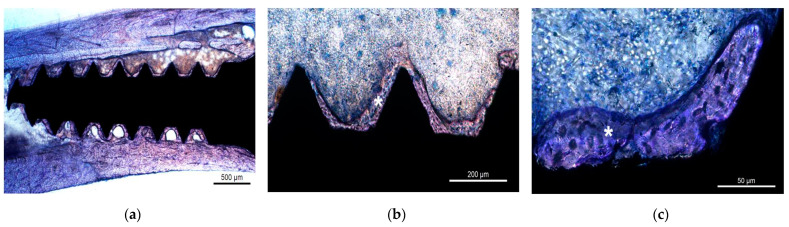
Histological photomicrographs of control samples showing the interface thirty days post implantation. Note the close contact between the surface of the biomaterial and lamellar bone (∗) (osseointegration) and the absence of an inflammatory response. Acrylic resin. Ground sections. Staining with toluidine blue 1%. Original magnification: (**a**) 25×, (**b**) 100×, and (**c**) 400×.

**Figure 8:**
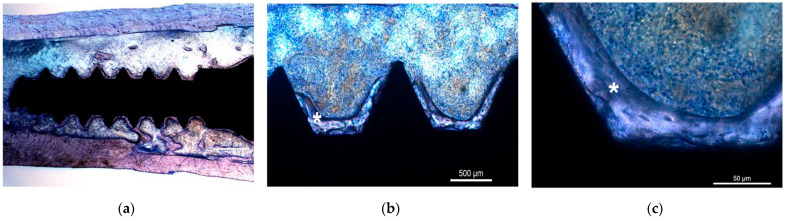
UNCD histological sections obtained thirty days post implantation. Note the lamellar bone (*) tissue in close contact with the UNCD surface and the absence of inflammatory infiltrate and multinucleated giant cells. Acrylic resin. Ground sections. Staining with toluidine blue 1%. Original magnification (**a**) 25×, (**b**) 100×, and (**c**) 400×.

Histologic examination of control and experimental samples revealed areas of lamellar bone in close contact with the surface of the implant (BIC, osseointegration) and areas of bone marrow in contact with the implant surface (myelointegration) thirty days post implantation (Figure 7a–c or Figure 8a–c). No inflammatory or multinucleated giant cells were detected.

### 3.6. Histomorphometry

**Figure 9:**
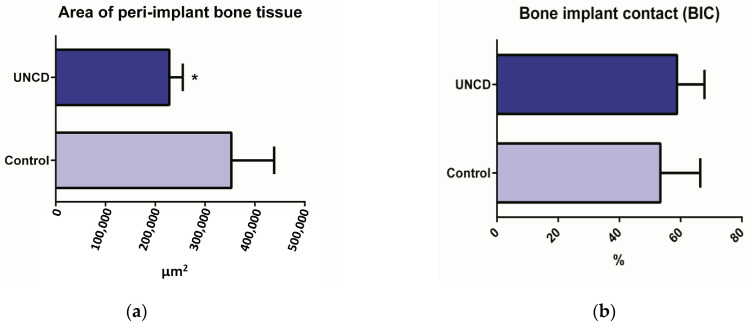
Histomorphometric study 30 days post implantation. (**a**) The area of peri-implant bone tissue was significantly greater in the C than in the UNCD group, (*) *p* < 0.05; (**b**) the percentage of BIC (osseointegration) was similar in both groups, *p* ≥ 0.05. The histograms show the mean ± SD, *p* < 0.05.

The histomorphometric study showed no statistically significant differences in the percentage of BIC (osseointegration) between groups (C: 53.40 ± 13% vs. UNCD: 58.82 ± 9%, *p* ≥ 0.05). The area of peri-implant bone tissue was significantly greater in the C group than in the UNCD group (C: 352932.7 ± 85.794 μm^2^ vs. UNCD: 228244.1 ± 26.800 μm^2^, *p* < 0.05) (Figure 9a,b).

## 4. Discussion

The potential toxicity and biological risks associated with ions/particles released as a result of biotribocorrosion of metallic implants is a public health concern that particularly affects patients carrying a metallic medical implant, whether orthopedic or dental, since these prostheses must remain inside the body over long periods of time, even decades [6,14,57,58]. Managing and controlling biotribocorrosion of a biomedical implant is therefore paramount from a biological, sanitary, metallurgic, economic, and social point of view. Furthermore, studies in the orthopedic and dental literature show that biotribocorrosion of titanium implants can lead to loss of osseointegration [7,57].

It remains unknown whether the presence of inflammation, which causes a decrease in pH, triggers corrosion processes [59,60], or whether corrosion processes, which result in the release of particles, trigger an inflammatory response. Whichever the case, the presence of macrophages loaded with Ti particles is a bioindicator of the occurrence of biotribocorrosion. Once the metallic particles are phagocytized by macrophages, a number of chemical proinflammatory mediators (cytokines, chemokines) are released and can, in turn, trigger a range of biological effects, including osteoclast activation, and thus favor bone resorption, inhibit osteoblasts from secreting bone matrix, and ultimately result in osteolysis [61,62,63].

There are also reports showing an immune response to Ti triggered by exposure to ions/particles released from an implant [18,19,64]. In a previous study, we evaluated biopsies of oral mucosa adjacent to implant cover screws. Positive immunohistochemical staining with antibodies anti-CD68 and anti-CD45RO confirmed the presence of macrophages and T lymphocytes associated with the metal particles [14]. This finding suggests an immune response mediated by cells and is in line with reports in the literature [65,66].

Histological examination performed in the present study revealed the presence of lamellar bone tissue in contact with the implants (osseointegration). Our histological results showed that UNCD-coated implants were well tolerated by the surrounding tissue and, like the non-coated implants, caused no inflammatory reaction.

The development of peri-implantitis triggered by the presence of metallic particles themselves and/or by their interaction or synergistic effect with periodontal pathogens [67,68,69] is another documented biological effect of Ti implants that is raising growing concern [67,68,69].

As to the carcinogenic potential of implants, there are scant reports on the possible development of malignant tumors associated with prosthetic structures in humans [20,21,22,70]. Nevertheless, there is no clear evidence that biotribocorrosion of Ti implants is not involved in carcinogenesis [71]. Of note, TiO_2_ was classified by the International Agency for Cancer Research as possibly carcinogenic to humans (Group 2B) [72].

Several mechanisms have been proposed to explain the possible association between malignant transformation and a metallic implant device. Features such as valence, particle concentration and size, and hypersensitivity have been proposed as potential factors [70]. Nevertheless, no direct cause-and-effect has been demonstrated in humans to date [70,71,73,74].

Specifically regarding titanium dental implants, 46 cases of squamous cell carcinoma [71,73], one osteosarcoma [20], and one plasmacytoma [21] in the vicinity of a Ti implant have been reported in the scientific literature in English. Some authors have suggested an association between the release of particles from a metallic implant and carcinogenic and mutagenic changes in the oral cavity [73,75]. In addition to the aforementioned studies, cases of breast, lung, and prostate metastases associated with dental implants have also been reported in the literature [71,73]. In a previous work, we reported [17] a case of intraosseous metastasis of kidney adenocarcinoma, which to our knowledge is the first report of this type of lesion close to a dental implant. It is important to point out that whereas there are no reports of a benign neoplasm developing in the vicinity of a titanium dental implant, our series included a case of peripheral cemento-ossifying fibroma.

According to Doak el at., evidence for the genotoxicity and carcinogenesis of carbon-based nanomaterials is accumulating, with a clear dependency on physicochemical features, but their long-term impact on human health has yet to be definitively established [76]. There are no reports in the literature suggesting a carcinogenic potential of the UNCD coating. We speculate that because the coating is a carbon-based material and carbon is a component of human tissue, it is highly biocompatible.

It is also known that trace metals can increase the physiological production of reactive oxygen species (ROS), which can lead to tissue damage in the absence of a compensatory increase in antioxidant species [77,78,79]. Previous experimental studies performed by our research group showed that TiO_2_ particles were transported in the blood via cells of the mononuclear phagocytic lineage and were deposited in organs with macrophagic activity such as the liver, spleen, and lungs and caused an increase in oxidative stress in lung and liver macrophages [13,80]. Toxicity studies performed by our research group allowed establishing a relation between the toxic effects of TiO_2_ particles and particle size. In line with a number of studies demonstrating that the smaller the particle, the greater its toxicity [81,82,83], our studies showed that superoxide anion generation was inversely proportional to particle size [84].

With the aims of preventing or limiting the release of TiO_2_ particles into the biological milieu and avoiding the likely adverse biological effects, UNCD coatings have been developed to optimize implant biocompatibility, improve osseointegration, and reduce the likelihood of biotribocorrosion.

Film adhesion is one of the crucial requirements for any type of coating for biomedical implants. In this regard, it has been demonstrated that UNCD coatings exhibit strong adhesion, even in situations where two UNCD-coated parts rub together due to a strong pushing force, as is the case of UNCD-coated mechanical pump seals [85].

Our group evaluated the in vivo biological effects and biokinetics of UNCD particles compared to the biological response to TiO_2_ nanoparticles in an experimental animal model [86]. The scant amount of UNCD deposits in the parenchyma of the analyzed organs, the absence of morphological alterations, and the lower oxidative inductions as compared to observations in TiO_2_-nanoparticle-exposed animals suggest that tissue response would be less aggressive or negligible in the event that the UNCD coating detached from the implant surface. These differences in biological response may be associated with the fact that UNCD coatings are made of carbon (C), a component of human DNA, cells, and molecules, which potentially makes them highly biocompatible coatings for medical implant devices [87].

Interestingly, tissue response to the UNCD coated implant was assessed in vivo in the present study [56]. The experimental model used here was developed by our research group [56]. The basic principle of the model is the osteogenic capacity of the rat tibia bone marrow, and it has been used to evaluate the influence of different systemic and local factors on tissue repair. The model provides an isolated microenvironment that is not exposed to microbial contamination or mechanical forces; this controlled environment allows evaluating tissue response to a specific condition, ruling out the interference of confounding variables. Our in vivo model allows implant immobilization. Radiographs confirmed that the biomaterial remained in the same place and position inside the tibial medullary cavity throughout the entire experiment, and thus served as a 3D osteoconductive scaffold.

Nanostructured surfaces have controlled physicochemical properties, including roughness, wettability, surface charge, and topography [26]. The UNCD coating had a surface with nanometric-scale structures and the typical grain size of 3–5 nm, enhancing implant surface interactions with ions, biomolecules, and cells and optimizing biocompatibility. Unlike conventional surface topographies, UNCD provides tailored surface nano-topography where osteoblasts recognize differences in nanometer range, favoring the osseointegration rate of UNCD implants [35,37].

The results of the present study allow concluding that UNCD has excellent biocompatibility properties and, though the percentage of BIC (osseointegration) was similar in UNCD-coated and control Ti micro-implants, its documented tribological properties would make it a superior implant coating material. Although the UNCD coating did not improve the percentage of osseointegration, it could protect against biotribocorrosion of the base material (titanium) and its ensuing deleterious biological effects.

Given the current surge in the use of nano-coatings, nanofilms, and nanostructured surfaces to enhance biocompatibility of biomedical implants, the results of the present study contribute valuable data for the manufacture of UNCD coatings as a new generation of superior dental implants. With regard to the above statement, it is relevant to consider recent R&D findings of a clinical trial conducted at a world-class clinic in Querétaro, Mexico, in 2018, involving implantation of UNCD-coated commercial Ti-6Al-4V dental implants in thirty-five patients. The results indicate that the biocompatible UNCD coatings will enable a new generation of dental implants that are superior to current metal-based dental implants. A recently published book chapter by our group [88] provides a detailed description of the materials and technological research and development of the UNCD-coated dental implants and of the results obtained in experimental animal studies and clinical trials.

## Data Availability

The data presented in this study are available on request from the corresponding author.
